# The toxicity of angiotensin converting enzyme inhibitors to larvae of the disease vectors *Aedes aegypti* and *Anopheles gambiae*

**DOI:** 10.1038/srep45409

**Published:** 2017-03-27

**Authors:** Zatul-’Iffah Abu Hasan, Helen Williams, Nur M. Ismail, Hidayatulfathi Othman, Gyles E. Cozier, K. Ravi Acharya, R. Elwyn Isaac

**Affiliations:** 1 Faculty of Medicine and Health Sciences, Universiti Sains Islam Malaysia, 13th Floor, Menara B, Persiaran MPAJ, Jalan Pandan Utama, Pandan Indah, 55100, Kuala Lumpur, Malaysia; 2 School of Biology, Faculty of Biological Sciences, University of Leeds, Leeds LS2 9JT, UK; 3 Liverpool Insect Testing Establishment, Department of Vector Biology, Liverpool School of Tropical Medicine, 1 Pembroke Place, Liverpool L3 5QA, UK; 4 School of Diagnostic & Applied Health Sciences, Faculty of Health Sciences, Universiti Kebangsaan Malaysia, Jalan Raja Muda Abd Aziz, 50300, Kuala Lumpur, Malaysia; 5 Department of Biology and Biochemistry, University of Bath, Claverton Down, Bath BA2 7AY, UK

## Abstract

The control of mosquitoes is threatened by the appearance of insecticide resistance and therefore new control chemicals are urgently required. Here we show that inhibitors of mosquito peptidyl dipeptidase, a peptidase related to mammalian angiotensin-converting enzyme (ACE), are insecticidal to larvae of the mosquitoes, *Aedes aegypti* and *Anopheles gambiae.* ACE inhibitors (captopril, fosinopril and fosinoprilat) and two peptides (trypsin-modulating oostatic factor/TMOF and a bradykinin-potentiating peptide, BPP-12b) were all inhibitors of the larval ACE activity of both mosquitoes. Two inhibitors, captopril and fosinopril (a pro-drug ester of fosinoprilat), were tested for larvicidal activity. Within 24 h captopril had killed >90% of the early instars of both species with 3^rd^ instars showing greater resistance. Mortality was also high within 24 h of exposure of 1^st^, 2^nd^ and 3^rd^ instars of *An. gambiae* to fosinopril. Fosinopril was also toxic to *Ae. aegypti* larvae, although the 1^st^ instars appeared to be less susceptible to this pro-drug even after 72 h exposure. Homology models of the larval *An. gambiae* ACE proteins (AnoACE2 and AnoACE3) reveal structural differences compared to human ACE, suggesting that structure-based drug design offers a fruitful approach to the development of selective inhibitors of mosquito ACE enzymes as novel larvicides.

The mosquitoes *Anopheles gambiae* and *Aedes aegypti* are some of the most deadly insects because of their effectiveness as vectors of malaria and a range of arboviruses, including yellow fever, dengue, chikungunya and zika. The close association of *Ae. aegypti* with urbanisation in tropical and sub-tropical countries and the ease of trans-global human travel and the mass migrations from war zones presents particular challenges in disrupting the cycle of arbovirus infections transmitted by *Ae. aegypti* in human populations^1^,^2^. The lack of effective vaccines and treatments for dengue, chikungunya and zika has focused attention on integrated vector control management based on environmental/cultural management, chemical and biological control^2^. The use of insecticides from different chemical classes is a key component of the integrated strategy against both *An. gambiae* and *Ae. aegypti*, but the ever increasing problem of insecticide resistance means that new compounds with different modes of action are urgently needed to replace chemicals that fail to control resistant mosquito populations^3^,^4^,^5^,^6^.

In our search for new insecticide targets that interfere with peptide hormone metabolism, we have previously identified a peptide-degrading zinc-metallopeptidase, known as angiotensin converting enzyme (ACE) from the role of the mammalian enzyme in the renin-angiotensin systems^7^,^8^,^9^, as a potential target for the development of inhibitors that disrupt insect reproduction. The dual role of the enzyme in the processing of the mammalian vasoconstrictor angiotensin II and the inactivation of the vasodilatory bradykinin led to the development of inhibitors of human ACE as anti-hypertensive drugs^10^,^11^,^12^. The first inhibitors were in fact natural proline-rich peptides (BPPs) with bradykinin-potentiating activity isolated from the venom of the snake *Bothrops jararaca*, but these lacked oral activity^13^,^14^,^15^.

Insect ACE, like the mammalian enzyme, is a promiscuous peptidase that cleaves dipeptides from the carboxyl end of oligopeptides and, in some instances, can cleave amidated di- or tri-peptides from substrates with an amidated carboxyl terminus, a common feature of insect neuropeptides^16^,^17^,^18^,^19^,^20^,^21^. Insect ACEs are generally soluble enzymes secreted from cells into the extracellular milieu, where like their mammalian counterparts they degrade peptides by sequential removal of dipeptides^16^,^22^,^23^,^24^. Much of our knowledge of the biochemistry and structural biology of insect ACE comes from studying the *Drosophila melanogaster* enzyme known as AnCE^21^,^25^. This peptidyl dipeptidase is strongly expressed as a glycosylated protein of 72 kDa in several tissues, including male reproductive tissues, the larval and adult midgut, larval trachea and adult salivary gland^26^,^27^. Other insect species also express ACE in reproductive tissues of both sexes, suggesting a broader physiological role for the enzyme in insect reproduction^19^,^28^,^29^,^30^,^31^,^32^,^33^,^34^,^35^,^36^. Insect ACE not only resembles the mammalian enzyme in its substrate specificity, but also in susceptibility to inhibitors such as captopril, lisinopril, fosinoprilat, enalapril and trandolaprilat, but apart from captopril these inhibitors can be far less potent towards insect ACE compared to mammalian ACE^22^,^23^. Nevertheless, ACE inhibitors can be acutely toxic to insects, which contrasts with their life-span extending properties in rodents^37^ and the nematode, *Caenorhabditis elegans*^38^. They have been useful in confirming important roles for ACE in reproduction, growth and development in several insect species^30^,^34^,^35^,^39^. In the mosquito *Anopheles stephensi*, females fed with ACE inhibitors in the blood meal or females mated with males supplied with ACE inhibitors in their sugar diet, lay significantly fewer eggs than normal^32^,^35^,^40^.

ACE inhibitors can also interfere with insect larval development as was shown by the stunting of larval growth of the tobacco hornworm, *Manduca sexta*, by injection of captopril, lisinopril and fosinoprilat into 4^th^ instar insects. Injection of the prodrug fosinopril into the same larval stages resulted in no weight gain and death within a few days. The injection of ACE inhibitors (captopril, lisinopril and enalapril) combined with the diuretic peptides (helicokinin I, II and III) into 5^th^ instar larvae of the tobacco budworm (*Heliothis virescens*) resulted in lethality, however, the inhibitors on their own were not toxic. In the present study we have asked the question whether mosquito larval stages, like the juvenile stages of some other insect species, are susceptible to ACE inhibitors. We now show that several synthetic ACE inhibitors are powerful blockers of the peptidyl dipeptidase activity found in a soluble fraction from both *Ae. aegypti* and *An. gambiae* larvae. The snake venom peptide BPP-12b, and the proline-rich trypsin-modulating oostatic factor (TMOF), which is toxic to *Ae. aegypti* larvae^41^, also inhibited the larval peptidase activity. When captopril and fosinopril (Fig. 1), two inhibitors with different modes of interaction with the ACE active site, were added to the rearing water high levels of larval mortality of were observed confirming the potential of insect ACE inhibitors as mosquito larvicides.

## Results and Discussion

### Inhibition of the soluble peptidyl dipeptidase (insect ACE) activity from whole larvae of *Ae. aegypti* and *An. gambiae*

**Figure 2:**
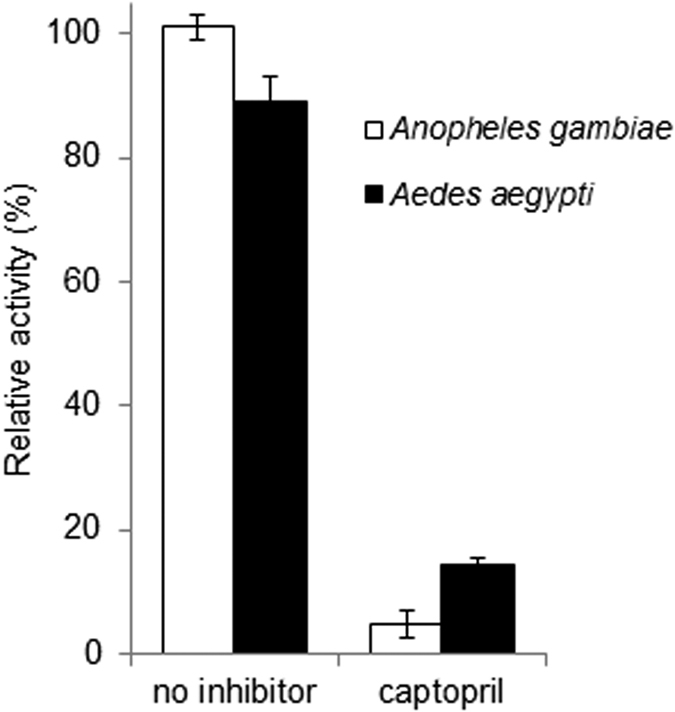
The relative activity of peptidyl dipeptidase (ACE) in a homogenate of mosquito larvae before and after high-speed centrifugation. Aliquots were removed for activity assay from the homogenate before and after centrifugation at 55,000 *g* for 1 h. The activity in the high-speed supernatant is expressed relative (%) to the activity of the homogenate prior to centrifugation. The results are expressed as the mean ± SEM. (n = 4).

**Figure 3:**
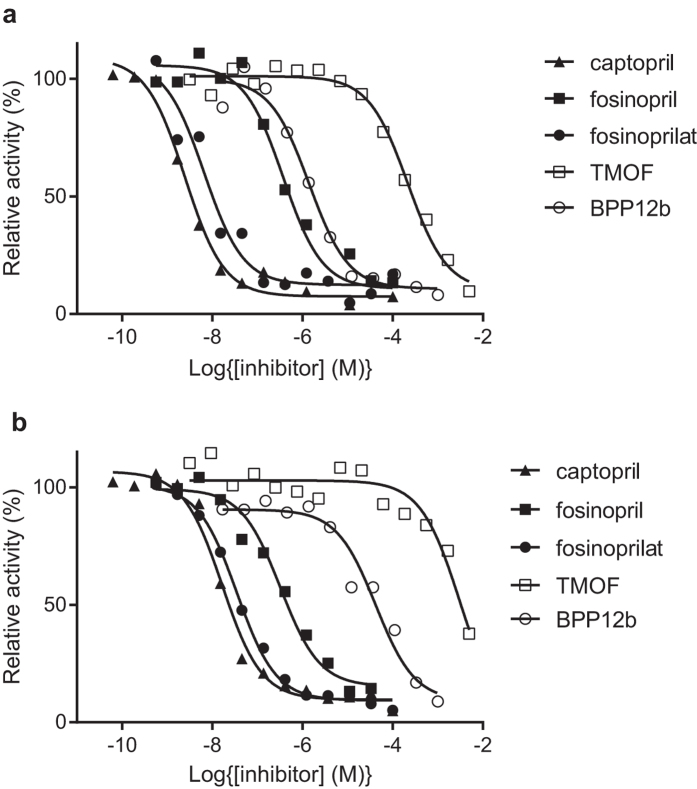
Inhibition of the peptidyl dipeptidase (ACE) activity of (**a**) *Ae. aegypti* and (**b**) *An. gambiae* larvae. Inhibition curves for captopril, fosinopril, fosinoprilat, PBB12b and TMOF were generated by assaying peptidyl dipeptidase activity of the high-speed supernatant prepared from a homogenate in the presence of different inhibitor concentrations using the assay described in the materials and methods section. Data is expressed relative to uninhibited activity.

Members of the insect ACE family of peptidyl dipeptidases are generally soluble secreted proteins, whereas mammalian ACEs are mainly membrane tethered. In order to ascertain whether the ACE activity of the mosquito larvae was soluble or membrane-bound, homogenates were prepared from 3^rd^ instar *Ae. aegypti* and *An. gambiae* and centrifuged at 55,000 g for 1 h to sediment cellular membranes. By measuring the peptidase activity before and after centrifugation, it was clear that the majority of the insect ACE remained in the high-speed supernatant and that most of the peptidase activity was inhibited by captopril (Fig. 2). The soluble enzyme from both mosquito species was then used to determine the relative potency of synthetic (captopril, fosinopril and fosinoprilat) and natural peptide (BPP-12b and TMOF) ACE inhibitors. Apart from the pro-drug fosinopril, all the inhibitors showed a greater degree of potency towards the *Ae. aegypti* activity, with captopril being the most potent (Fig. 3). For the enzyme prepared from both species, fosinopril was weaker than the non-esterified fosinoprilat in inhibiting the activity. The snake venom peptide BPP-12b and the mosquito peptide TMOF both inhibited mosquito ACE activity, but were much less potent than the synthetic compounds with BPP-12b having IC_50_ values two orders of magnitude lower than those of TMOF. Interestingly, both natural peptides were stronger inhibitors of the *Ae. aegypti* ACE compared to *An. gambiae* (Table 1).

### The toxicity of ACE inhibitors to larval instars of *Ae. aegypti* and *An. gambiae*

**Figure 4:**
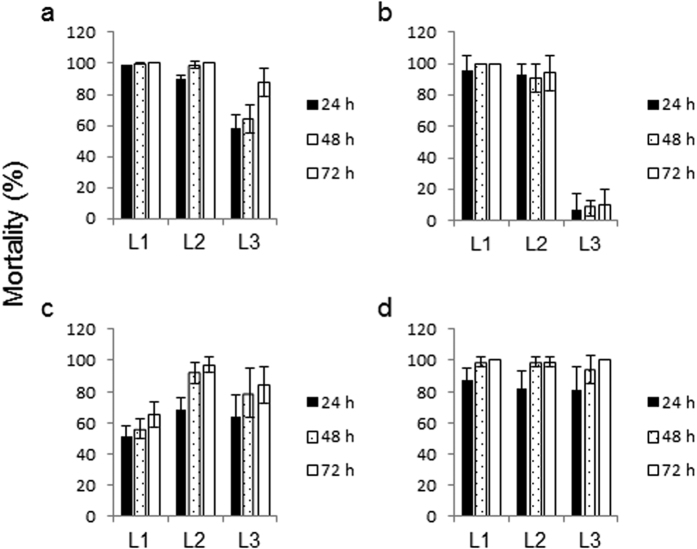
Larvicidal activity of captopril and fosinopril against *Ae. aegypti* and *An. gambiae* larvae. Larvae of *Ae. aegypti* (**a** and **c**) and *An. gambiae* (**b** and **d**) were placed in water containing food with either 5 mM captopril (**a** and **b**) or 5 mM fosinopril (**c** and **d**). Mortality was assessed every 24 h. In the absence of inhibitor all larvae survived and successfully developed into pupae. Data are expressed as the mean ± SD (n = 6).

The juvenile part of the mosquito life-cycle involves four larval instars (L1, L2, L3 and L4) of rapid growth and three larval moults before the transition to a pupa and complete metamorphosis to the adult. We investigated the effect of captopril and fosinopril on the survival of three larval stages (L1, L2 and L3) of both *Ae. aegypti* and *An. gambiae* by adding the chemicals to the water environment with the larval food followed by assessing mortality after 24, 48 and 72 h. High mortality was recorded for three larval instars (L1, L2 and L3) of *Ae. aegypti* after the first 24 h of treatment with captopril and by 48 h essentially all L1 and L2 insects were dead (Fig. 4a). The L3 instars were slightly more resistant to captopril, nevertheless by 72 h mortality of these insects had reached around 90%. Very similar results were obtained when L1 and L2 larvae of *An. gambiae* were treated in an identical manner with captopril (Fig. 4b). The L3 insects were, however, more resistant compared to *Ae. aegypti* at the same stage of development. Even after 72 h only 10% of the *An. gambiae* L3 larvae had succumbed to the inhibitor.

High levels of larval mortality were also recorded for *Ae. aegypti* treated with fosinopril (Fig. 4c). L1 larvae however appeared to be more resistant compared to the other two instars even after 72 h of treatment, which might reflect a lower metabolic conversion of the pro-drug to the active inhibitor fosinoprilat. On the other hand fosinopril showed high levels of toxicity to all three instars of *An. gambiae* with over 80% mortality within 24 h and over 95% mortality by 48 h of treatment (Fig. 4d).

We have shown previously that ACE inhibitors fed to adult mosquitoes can have profound effects on female fecundity, suggesting that the mosquito peptidyl dipeptidase is a potential target for the development of novel mosquito control chemicals^32^,^35^,^40^. We have now provided data supporting this view from a study of the effects of ACE inhibitors on the development and survival of larval instars of both *Ae. aegypti* and *An. gambiae*. Captopril was one of the first generation of orally active ACE inhibitors and is also the smallest, interacting with only two enzyme sub-sites (S1′ and S2′). These sub-sites have been labelled based around the peptide bond that is cleaved such that the pockets that bind the dipeptide that is released by the enzyme are labelled S_1_′ and S_2_′ sub-sites, and the dipeptide itself is labelled P_1_′ and P_2_′. The rest of the peptide is labelled P_1_, P_2_, etc, which bind in subsites S_1_, S_2_, etc, with the peptide being cleaved between residues P_1_ and P_1_′. Key to the design of captopril was the inclusion of a sulfhydryl group that interacts with the active site zinc. Other ACE inhibitors can be of a larger design enabling interaction with more than two enzyme sub-sites and may have a different zinc-binding ligand. In the case of fosinoprilat, a hydroxyphosphinyl group interacts with the zinc and this larger molecule binds at three sub-sites (S1, S1′ and S2′) of the enzyme. It is possible that off-target interactions are responsible or contribute to the toxicity observed in this study, however the fact that two ACE inhibitors from different chemical classes show similar toxic effects suggests that mosquito ACE is indeed the likely primary target. The concentration of inhibitor required for toxicity is much higher than the *in vitro* potency as inhibitors of the larval ACE, indicating some limited availability of the chemicals to the *in vivo* enzyme. We expect that the water soluble inhibitors are taken up orally with the food rather than through the cuticle. The mosquito larvae feed by filtering out organic particulate matter from the water, a mode of feeding that will limit ingestion of the inhibitors via the oral route. The pro-drug fosinopril has greater hydrophobicity to improve bioavailability and undergoes metabolic de-esterification to fosinoprilat, the active inhibitor. These chemical properties and the requirement that fosinopril undergoes metabolic activation are likely to be important factors in determining the toxicity of the inhibitor to mosquito larvae.

We tested BPP-12b, a member of the BPP family of peptides originally isolated from the venom of the pit viper, *Bothrops jararaca*, as an inhibitor of mosquito peptidyl dipeptidase and showed that the proline-rich peptide was a powerful natural inhibitor of the activity in both species. The presence of the Pro-Pro sequence at the C-terminus and a pyroglutamate at the N-terminus protects these peptides from metabolic degradation by exo-peptidases^7^,^42^. TMOF is a mosquito peptide isolated from the ovaries of adult *Ae. aegypti* that is also proline rich and therefore it was not surprising that it too inhibits mosquito larval peptidase activity, albeit with reduced potency relative to BPP-12b. TMOF is involved in regulating egg development and blood digestion following a blood meal^43^. It appears to work by inhibiting the biosynthesis of midgut trypsin and not by inhibition of the protease activity^41^. TMOF also inhibits trypsin biosynthesis in larval instars and has been used as a larvicide against *Ae. aegypti*. It is most effective when used in combination with δ-endotoxins from *Bacillus thuringiensis*^44^. TMOF is not a particularly potent inhibitor of larval mosquito ACE, nevertheless this inhibition might contribute in a minor way to the toxicity against *Ae. aegypti* larvae.

**Figure 5:**
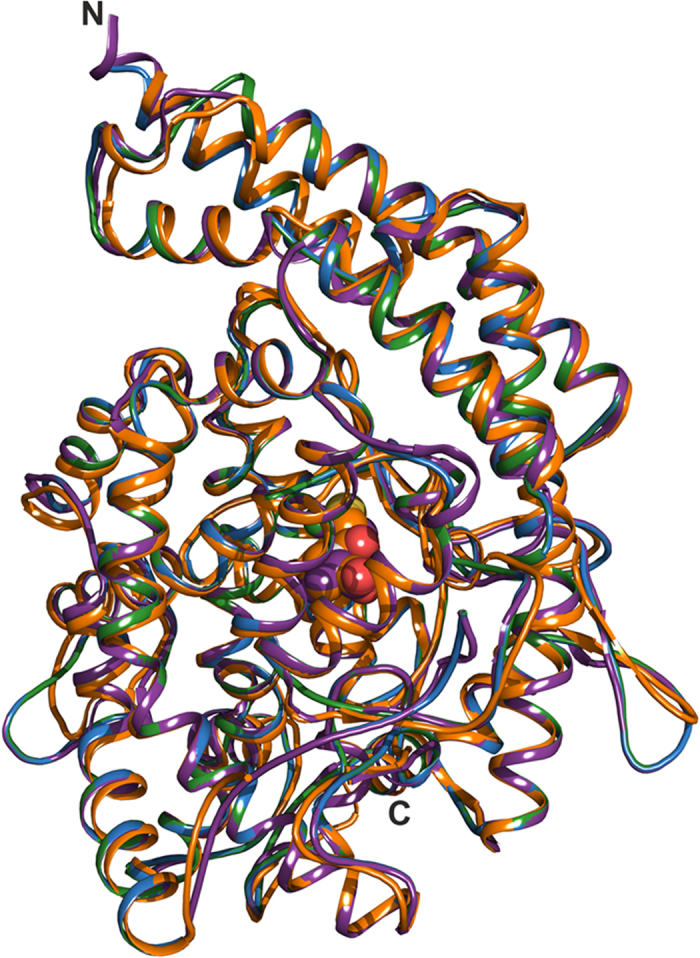
Cartoon representation overlay of human tACE- and AnCE-captopril complex crystal structures with modelled AnoACE2 and AnoACE3. tACE is shown in orange, AnCE in purple, AnoACE2 in blue and AnoACE3 in green. The captopril ligands are shown as spheres. The figure was generated using Pymol^59^.

The insect ACE gene family has expanded greatly in mosquitoes to nine (AnoACEs 1–9) and there is evidence that four *An. gambiae* genes (AnoACE2, AnoACE3, AnoACE7 and AnoACE9) are expressed in larval stages. AnoACE7 and AnoACE9 are predicted to have a C-terminal hydrophobic sequence that can form a membrane anchor^45^. Since the majority of the peptidyl dipeptidase activity in homogenates of larvae is soluble in nature, it seems likely that the activity results from expression of one or both of AnoACE2 and AnoACE3. Homology models for AnoACE2 and AnoACE3 were prepared using SWISS-MODEL^46^,^47^,^48^ with the crystal structure of *Drosophila melanogaster* ACE homologue (also known as AnCE) (PDB code 2 × 8Y)) used as the template (Fig. 5)^49^.

Our extensive knowledge from high resolution structural studies on how inhibitors interact with both the N- and C-domains of human ACE and insect ACE^25^,^49^,^50^,^51^,^52^,^53^,^54^,^55^,^56^,^57^,^58^ has revealed the molecular basis of binding features of the inhibitors and highlights differences in the inhibitor-enzyme interaction of insect ACE compared with the active sites of the human enzyme. Using the AnoACE homology models and amino acid sequence alignment, it is possible to compare different ACE proteins to look for residue and environment differences in both AnoACE2 and AnoACE3, which could be targeted to create specific inhibitors against these enzymes. Therefore the protein residues forming the binding sub-sites (S_2_′, S_1_′ S_1_ and S_2_) for the side chains of the region of the peptide (P_2_′, P_1_′, P_1_ and P_2_) spanning the catalytic site were compared between the different ACE proteins (Table 2). This shows that there are some residues in all four sub-sites that are conserved between human testicular ACE (tACE), which corresponds to the C-domain of human somatic ACE, and the insect ACEs (AnCE, AnoACE2 and AnoACE3). These are residues that could be targeted to increase potency of inhibitors as they are likely to be important in ligand binding, but this will not enhance specificity for AnoACE2 or AnoACE3. There are some residues in tACE that are conserved in either one, or both of the AnoACE proteins, but not in AnCE. These residues would be interesting to target for developing AnCE specific inhibitors. There are also some residues which are identical between AnCE and one, or both of the AnoACE proteins, but not tACE. This provides information for developing a broader insect specific inhibitor. Of particular interest to this study, some residues are unique to either one or both of the AnoACE proteins, and targeting these differences are the most likely route for the development of AnoACE2 and AnoACE3 specific inhibitors. These variations of the sub-sites have already been shown to give rise to different substrate and inhibitor specificities between the different ACE homologues. For example, differences in the S_2_ sub-site between the N- and C-domains of human somatic ACE have been identified as the cause of certain substrate and inhibitor specificity differences. In particular the substitution of F391 in the C-domain, to Y369 in the N-domain, has been attributed to C-domain selectivity of RXPA380 (see recent review ref. 25). Both AnoACE2 and AnoACE3 also have a tyrosine in this position, but in addition AnoACE2 has another potentially significant change in the S_2_ sub-site with G403 replacing E403 of tACE (Table 2). This suggests that perhaps the S_2_ sub-site maybe important for creating an AnoACE2 specific inhibitor, which not only lacks the charge of the E403 found in tACE, but also has a larger binding pocket due to significantly smaller glycine residue.

**Figure 6:**
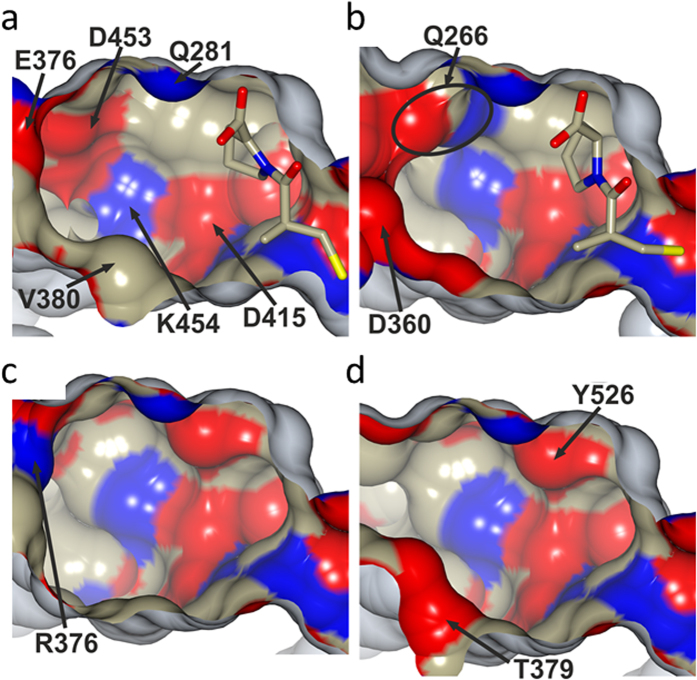
Surface representation looking into the S_1_’ and S_2_’ sub-sites cavity of (**a**) human tACE-captopril complex, (**b**) AnCE-captopril complex, (**c**) AnoACE2 model and (**d**) AnoACE3 model. The surface is coloured based on atom type (nitrogen in blue, oxygen in red and carbon in beige) and captopril ligands are shown as sticks. Selected residues are labelled to highlight differences between proteins. The figure was generated using CCP4mg^60^.

There are additional notable differences between the AnoACE homologues and human tACE in the S_2_′ sub-site, and these include changes in size, shape and charge of the side-chains involved. These differences will cause a variation in the size, shape and localised charge of this sub-site between the different proteins, highlighted by the variations shown on the inner protein surface of this void (Fig. 6). Therefore the S_2_′ sub-site would be of particular interest for AnoACE specific inhibition. Additional non-conserved amino acid substitutions are found in the S_1_ and S_1_′ sub-sites. Although less than observed in the S_2_′ sub-site, they could still result in significant charge changes at the binding site that will in turn influence inhibitor binding.

This level of detail makes AnoACE an attractive target for applying structure-based drug design to developing potent and, importantly, highly selective inhibitors which could be used as insecticides. These compounds would need to be presented to the larvae of *Ae. aegypti* and *An. gambiae* in a particulate form to maximise oral activity in these filter feeders.

## Methods

### Chemicals

Fosinopril was purchased from Generon Ltd., Maidenhead, Berkshire, U.K. TMOF and BPP-12b was purchased from Biomatik USA, LLC, Wilmington, Delaware, USA. All other ACE inhibitors were purchased from Sigma-Aldrich Company Ltd., Poole, Dorset, U.K. The ACE substrate Abz-Phe-Arg-Lys(Dnp)-Pro-OH (Abz-FRK(Dnp)-P) was from Enzo Life Sciences (UK) Ltd, Exeter, U.K.

### Mosquito culture

*Ae. aegypti* (Jinjang, Kuala Lumpur strain) and *An. gambiae* (Kisumu, Kenya strain) were reared in laboratory insectaries and using standard culture methods at the Faculty of Medicine and Health Sciences, Universiti Sains Islam Malaysia and the Liverpool School of Tropical Medicine, respectively.

### Preparation of mosquito enzyme

Larvae (15, 3^rd^ instar) were washed in distilled water 5 times for 10 minutes and final wash for 1 hour at room temperature before storage at −20 °C until required. Homogenate was prepared using a glass homogeniser (Jencons, East Grinstead, U.K.), containing 0.5 ml of 100 mM HEPES buffer pH 7.5, 50 mM NaCl and 10 μM ZnCl_2_, and 20 up and down strokes of the pestle. A soluble fraction was prepared from the homogenate using a Beckman Optima™ MAX bench-top ultracentrifuge and TLA110 rotor (Beckman Instruments Inc, Palo Alto, Ca, U.S.A.) operating at 55,000 g (4 °C) for 1 h. Aliquots of the homogenate were taken prior to centrifugation to determine the relative distribution of the peptidase activity before and after centrifugation.

### Assay of peptidyl dipeptidase activity

Rates of hydrolysis of the quenched fluorogenic substrate Abz-FRK(Dnp)-P by *Ae. aegypti* and *An. gambiae* enzymes were performed at 20 °C in 96-well black plastic plates (Corning Life Sciences, High Wycombe, U.K.) using a FLUOstar Omega (BMG LABTECH GmbH, Offenburg, Germany) with λ_ex_ at 340 nm and λ_em_ set at 430 nm). The reaction was started by adding 1 μl of 5 mM Abz-FRK(Dnp)-P in dimethyl sulfoxide) to the enzyme in 200 μl of 100 mM HEPES buffer pH 7.5, 50 mM NaCl and 10 μM ZnCl_2_. For studying the effect of ACE inhibitors, enzyme was pre-incubated with inhibitor for 10 min prior to the addition of substrate.

### Larvicidal activity of ACE inhibitors on *Ae. aegypti* and *An. gambiae* larvae

Larvicidal testing was performed in 24-well plastic plates (Becton Dickinson Labware, NJ, USA) using 20 *Ae. aegypti* and *An. gambiae* larvae of each L1, L2 and L3 stage in distilled water. Inhibitors dissolved in water were added separately to each well containing larvae to give a final concentration of 5 mM with Brewer’s yeast as the food source. Natural mortality was assessed by carrying out a parallel study with the same volume of water and number of larvae in the absence of ACE inhibitor. Mortality in each well was recorded after 24, 48 and 72 h of exposure.

## Additional Information

**How to cite this article:** Abu Hasan, Z.-I. *et al*. The toxicity of angiotensin converting enzyme inhibitors to larvae of the disease vectors *Aedes aegypti* and *Anopheles gambiae. Sci. Rep.* **7**, 45409; doi: 10.1038/srep45409 (2017).

**Publisher's note:** Springer Nature remains neutral with regard to jurisdictional claims in published maps and institutional affiliations.

## Figures and Tables

**Figure 1:**
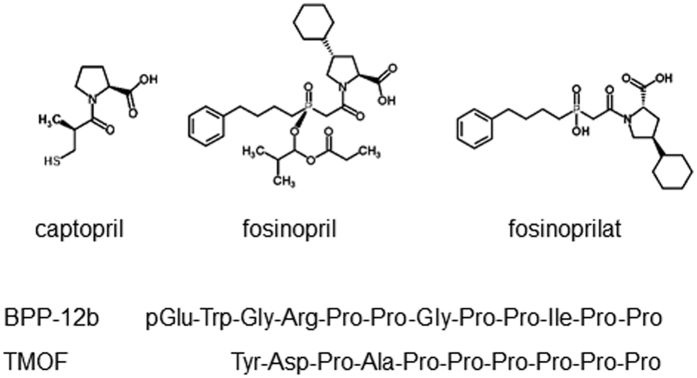
Chemical structures of inhibitors of mosquito larval peptidyl dipeptidase activity.

**Table 1:** IC_50_ values for the inhibition of larval peptidyl dipeptidase (ACE) activity.

Inhibitor	Aedes aegypti	Anopheles gambiae
	IC_50_ (M)	IC_50_ (M)
Captopril	2.47E-09	1.67E-08
Fosinopril	3.73E-07	3.26E-07
Fosinoprilat	6.61E-09	3.52E-08
TMOF	2.16E-04	3.10E-03
BPP-12b	1.40E-06	4.23E-05

**Table 2:** Residues involved in forming the sub-sites close to the catalytic site for human tACE, AnCE and modelled AnoACE2 and AnoACE3.

Sub-site	tACE	AnCE	AnoACE2	AnoACE3
S_1_	**E143**	**E124**	**E140**	**Q139**
	*V351*	*V335*	*V351*	*V350*
	*S355*	*S339*	*S355*	*S354*
	*K368*	*K352*	*K368*	*K367*
	**F512**	***Y496***	***Y512***	***Y511***
	**S516**	***A500***	***A516***	***A515***
	*V518*	*V502*	*V518*	*V517*
S_2_	*A356*	*A340*	*A356*	*A355*
	**F391**	**F375**	**Y391**	**Y390**
	**E403**	**T387**	**G403**	**E402**
	*H410*	*H394*	*H410*	*H409*
	*R522*	*R506*	*R522*	*R521*
S_1_′	**E162**	***D146***	***D162***	**E161**
	**T166**	**E150**	**T166**	**T165**
	*W279*	*W263*	*W279*	*W278*
	*A354*	*A338*	*A354*	*A353*
	**D377**	**Q361**	**E377**	**D376**
	**V380**	***T364***	**V380**	***T379***
	*E384*	*E368*	*E384*	*E383*
S_2_′	*N277*	*N261*	*N277*	*N276*
	*Q281*	*Q265*	*Q281*	*Q280*
	**T282**	**Q266**	**T282**	**T281**
	**S284**	**S268**	**D284**	**D283**
	**E376**	**D360**	**R376**	**E375**
	**V379**	***F363***	***F379***	***F378***
	*D415*	*D399*	*D415*	*D414*
	**A418**	***S402***	***S418***	***S417***
	**D453**	**D437**	**T453**	**A452**
	*K454*	*K438*	*K454*	*K453*
	*F457*	*F441*	*F457*	*F456*
	**F527**	**F511**	**Y527**	**Y526**

Completely conserved residues are highlighted in *italics*, residues identical to tACE in **bold**, residues identical to AnCE are in bold italics, and unconserved residues are in underlined bold font.
